# Trend in global burden attributable to low bone mineral density in different WHO regions: 2000 and beyond, results from the Global Burden of Disease (GBD) study 2019

**DOI:** 10.1530/EC-23-0160

**Published:** 2023-09-14

**Authors:** Nekoo Panahi, Sahar Saeedi Moghaddam, Noushin Fahimfar, Negar Rezaei, Mahnaz Sanjari, Mohammad-Mahdi Rashidi, Parnian Shobeiri, Bagher Larijani, Afshin Ostovar

**Affiliations:** 1 Metabolic Disorders Research Center, Endocrinology and Metabolism Molecular-Cellular Sciences Institute, Tehran University of Medical Sciences, Tehran, Iran; 2 Osteoporosis Research Center, Endocrinology and Metabolism Clinical Sciences Institute, Tehran University of Medical Sciences, Tehran, Iran; 3 Non-Communicable Diseases Research Center, Endocrinology and Metabolism Population Sciences Institute, Tehran University of Medical Sciences, Tehran, Iran; 4 Kiel Institute for the World Economy, Kiel, Germany; 5 Department of Epidemiology and Biostatistics, School of Public Health, Tehran University of Medical Science, Tehran, Iran; 6 Endocrinology and Metabolism Research Center, Endocrinology and Metabolism Clinical Sciences Institute, Tehran University of Medical Sciences, Tehran, Iran

**Keywords:** low BMD, osteoporosis, GBD, burden, DALY

## Abstract

**Background:**

We aimed to document the current state of exposure to low bone mineral density (BMD) and trends in attributable burdens between 2000 and 2019 globally and in different World Health Organization (WHO) regions using the Global Burden of Disease (GBD) study 2019.

**Methods:**

We reviewed the sex-region-specific summary exposure value (SEV) of low BMD and the all-ages numbers and age-standardized rates of disability-adjusted life years (DALYs), years lived with disability (YLDs), years of life lost (YLLs), and deaths attributed to low BMD. We compared different WHO regions (Africa, the Eastern Mediterranean Region, Europe, Region of the Americas, Southeast Asia, and Western Pacific), age categories, and sexes according to the estimates of the GBD 2019 report.

**Results:**

The global age-standardized SEV of low BMD is estimated to be 20.7% in women and 11.3% in men in 2019. Among the WHO regions, Africa had the highest age-standardized SEV of low BMD in women (28.8% (95% uncertainty interval 22.0–36.3)) and men (16.8% (11.5–23.8)). The lowest SEV was observed in Europe in both women (14.7% (9.9–21.0)) and men (8.0% (4.3–13.4)). An improving trend in the global rate of DALY, death, and YLL was observed during 2000–2019 (−5.7%, −4.7%, and −11.9% change, respectively); however, the absolute numbers increased with the highest increase observed in global YLD (70.9%) and death numbers (67.6%). Southeast Asia Region had the highest age-standardized rates of DALY (303.4 (249.2–357.2)), death (10.6 (8.5–12.3)), YLD (133.5 (96.9–177.3)), and YLL (170.0 (139–197.7)).

**Conclusions:**

Overall, the highest-burden attributed to low BMD was observed in the Southeast Asia Region. Knowledge of the SEV of low BMD and the attributed burden can increase the awareness of healthcare decision-makers to adopt appropriate strategies for early screening, and also strategies to prevent falls and fragility fractures and their consequent morbidity and mortality.

## Background

Osteoporosis is a degenerative age-related disease characterized by reduced bone mass and deteriorated bone microstructure and predispose individuals to fragility fractures (1). Bone strength is determined by its quantity and quality. However, currently, bone mineral density (BMD) obtained by dual-energy X-ray absorptiometry (DXA) is used as the standard for the diagnosis of osteoporosis (2). Low BMD is a risk factor for osteoporotic fractures defined as fractures caused by low-impact trauma and each standard deviation decrease in femoral neck BMD is associated with a two- to three-fold increase in the risk of fracture (3) with the consequent morbidity and mortality and health expenditures (4, 5). Osteoporosis is a silent disease until a fragility fracture occurs, sometimes with severe long-term consequences. For instance, a hip fracture has a considerable impact on the abilities, function, quality of life, and health expenditures of elderly individuals (6) and leads to a significant loss of healthy life years mostly due to disability in elderly people (7). Epidemiology and burden of osteoporosis are among the main foci of highly cited papers in the field of osteoporosis (8). The rate of fractures and the consequent burden is increasing in parallel to the population aging and increase in life expectancy which may affect developing countries more than developed ones, making it a serious public health issue (9). Therefore, current knowledge of the epidemiological pattern and the burden is mandatory to decide on strengthening the primary prevention measures.

Based on the Global Burden of Disease (GBD) study 2019 data, recent literature demonstrated that apart from North America, the global prevalence of low BMD decreased from 1990 to 2019 with higher incidence and mortality rates observed in countries with low socio-demographic index (SDI) (10). Also despite the higher prevalence of low BMD in women, men were exposed to higher rates of disability-adjusted life year (DALY) and mortality, especially in countries with middle-to-high SDI (11). The current state of osteoporosis and the attributable burden, as well as the recent trend, has not been visualized globally according to World Health Organization (WHO) regions recently. In this regard, we followed the methodology used in the GBD study 2019 which estimates the incidence and prevalence of diseases and injuries, the summary exposure value (SEV) of risk factors, and the attributable burden in different countries and territories (12, 13). Osteoporosis has not been considered a disease in GBD studies; therefore, we used low BMD, considered a risk factor in these studies as the nearest surrogate and proxy. Since health policymaking at the international level is usually defined and based on WHO regions in the present study, we aimed to document the SEV of low BMD at the global level and in different WHO regions by sex. We also aimed to evaluate the trends in the attributable burdens between 2000 and 2019 globally and in different WHO regions using the results of the GBD study 2019.

## Methods

### Study design

In the present study, we reviewed the sex-region-specific SEV of low BMD and the all-ages numbers and age-standardized burden rates attributed to low BMD including DALYs, years lived with disability (YLDs), years of life lost (YLLs), and deaths using the estimates of the GBD 2019 report. We compared different WHO regions, age categories, and sexes as well. The GBD study 2019 estimates the incidence and prevalence of 369 diseases and injuries, the SEV of 87 risk factors, and the attributable burden including mortality, YLLs, YLDs, and DALYs in 204 countries and territories (12, 13). The estimated outcome measures in the GBD 2019 study can be obtained at the global level, regionally, and for different countries and territories.

Low BMD is considered a risk factor in the GBD study. Categories of risk factors include environmental/occupational, behavioral, and metabolic in the order of exposure value. The latter (metabolic) category includes high fasting plasma glucose, high low-density lipoprotein cholesterol (LDL) , high systolic blood pressure (SBP), high body mass index (BMI), low BMD, and kidney dysfunction (13). The burden data (data on DALYs, YLDs, YLLs, and mortality) for low BMD and other diseases, injuries, and risk factors are publicly available online by region, country, year, age, and sex (https://vizhub.healthdata.org/gbd-results/).

### Data sources

All of the data described and analyzed in this study are obtained from the GBD 2019 study. The data sources used in the GBD 2019 study include scientific literature, estimate, and surveys. Representative, population-based surveys that reported quantitative BMD of the femoral neck region, measured by DXA in g/cm^2^ were included in the GBD 2019 study. In some surveys, the mean BMD was reported in stratified groups rather than for the whole sample, the stratified means were aggregated to obtain a total mean BMD at the population level for age/sex categories (13). Overall, 168 surveys from 49 countries were included in the GBD 2019 study (13).

### Variables

In the GBD 2019 study, low BMD was defined using the theoretical minimum risk exposure level (TMREL); i.e., by the difference between the BMD of a population and the age-sex-specific 99th percentile of BMD from the National Health and Nutrition Examination Survey (NHANES) study as the reference population. In the GBD 2019, the attributed low BMD burden refers to the burden of injury resulting from low BMD; however, in the present study we use low BMD as a risk factor and the nearest surrogate to examine the burden of osteoporosis.

SEV is a risk-weighted prevalence and is measured on a scale of 0–100 accounting for the whole population exposed to a minimum (no excess risk) and maximum risk (highest level of risk), respectively (13).

The burden attributable to low BMD exposure was measured by DALYs, YLDs, YLLs, and deaths by sex and region and was estimated for women and men 40 years and older (12). DALYs is a population health metric and refers to the lost years of healthy life and is a combination of YLLs (lost years of life due to earlier death) and YLDs (years of life spent in disability adjusted by its weight) (14).

YLLs = number of deaths × global standard life expectancy at the age at which death occurs; YLDs = number of prevalent cases × weight of disability; DALYs = YLLs + YLDs

WHO regions include Africa, the Eastern Mediterranean Region (EMR), Europe, Region of the Americas, Southeast Asia, and Western Pacific.

### Statistical analysis

In the GBD 2019 study, the GBD standard population was used to calculate the age-standardized rates with the direct method (12). Mean BMD was modeled in DisMod-MR 2.1 as a single ‘continuous’ parameter model by age and sex, and all GBD locations for the years 1990–2019. The covariates used in the BMD DisMod-MR meta-regression model in GBD 2019 study included age-standardized total physical activity (MET-min/week), tobacco consumption (cigarettes per capita), mean BMI, and unadjusted calcium intake (grams) at the country level (13).

This paper describes data on age-standardized SEV of low BMD at the global level and in different WHO regions with their 95% uncertainty intervals (95% UI). It also presents the all-age and age-standardized burden per 100,000 population at a global level as well as an age-standardized burden in different WHO regions. In addition, this paper visualizes time trends of the burden attributable to low BMD through 2019 globally and in different WHO regions. We also present the difference in the attributable burden between different age categories.

Details of the GBD 2019 burden of diseases and attributed burden to risk factors methodology have been presented in previous publications (12, 13).

## Results

### Summary exposure value

**Figure 1:**
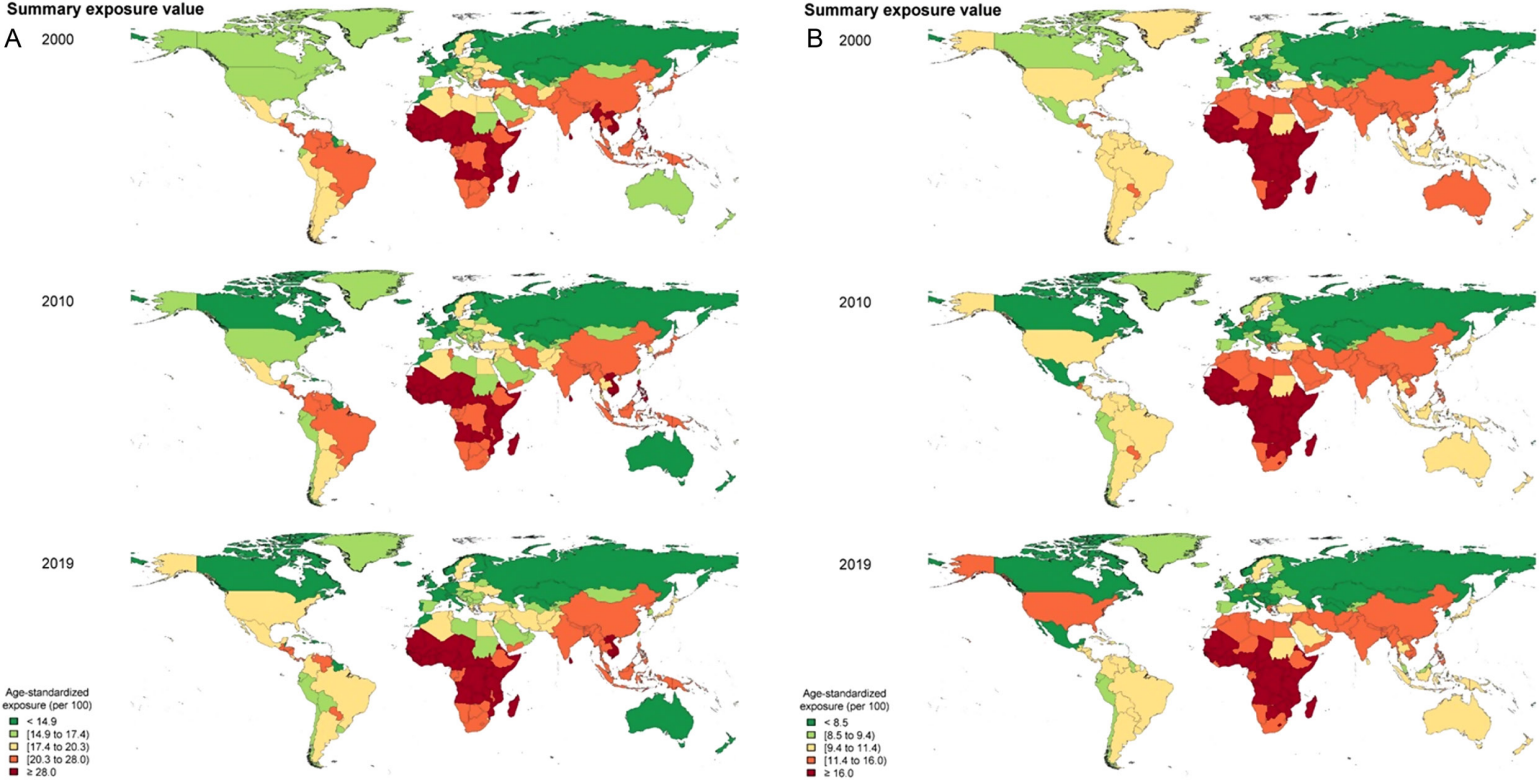
World distribution of exposure to low bone mineral density at the country level in (A) women and (B) men.

The age-standardized SEV to low BMD had a declining trend globally (16.7 (12–23) in 2000 and 16.3 (11.4–22.6) in 2019) and in all WHO regions during 2000–2019 except for the regions of America which increased from 14.3 (9.7–20.7) to 14.7 (9.9–21.1). The worldwide distribution of exposure to low BMD for people aged 40 years and more for the years 2000, 2010, and 2019, by sex, is presented at the country level (Fig. 1) and by WHO region (Supplementary Fig. 1, see section on supplementary materials given at the end of this article). Women had a higher SEV compared to men globally (20.7 (15–27.3) and 11.3 (7–17.6), respectively, in 2019) and in all WHO regions. The highest SEV of low BMD was detected in African women (28.8, 95% UI (22.0–36.3)), followed by women of Southeast Asia regions (22.9 (17.1–29.7)) in the year 2019 (Table 2, right column). In all, the European region showed the lowest SEV of low BMD in both women (14.7 (9.9–21)) and men (8 (4.3–13.4)). The rank of different WHO regions regarding SEV did not change from 2000 to 2019 when considering both women and men. However, in women, Southeast Asia and the regions of America showed a higher rank in 2019 (second and fourth, respectively) compared to 2010 (third and sixth rank, respectively). In men, EMR ranked higher in 2010 (second) compared to 2000 (third) and remained the second rank afterward (Supplementary Fig. 1).

### Attributable burden

#### Disability-adjusted life years

DALYs attributable to low BMD in both sexes increased from 10.6 million (8.7–12.6) in 2000 to 16.6 million (13.5–20) in 2019 at the global level (percent change of 57.0%; Table 1). However, the age-standardized rate of global DALYs showed an improving trend from 219.3 (179.9–260.8) per 100,000 in 2000 to 206.8 (167.9–248.7) in 2019 (percent change of −5.7%; Table 1). Burden values of low BMD in 2000, 2010, and 2019 at the global level and by WHO region are presented in detail in Tables 1 and 2, respectively.

**Table 1:** Global burden of low bone mineral density in 2000, 2010, and 2019 with percent change from 2000 to 2019.

Sex	Age, Metric	YLLs				YLDs				Deaths				DALYs			
		2000	2010	2019	Percent change (%)^a^	2000	2010	2019	Percent change (%)^a^	2000	2010	2019	Percent change (%)^a^	a2000	2010	2019	Percent change (%)^a^
Both	All ages number	5,563,744 (4,775,117 to 6,036,937)	7,023,744 (5,885,522 to 7,723,568)	8,026,949 (6,699,941 to 9,036,379)	44.3%	5,042,826 (3,602,050 to 6,800,208)	6,305,141 (4,491,475 to 8,502,776)	8,620,517 (6,115,780 to 11,640,097)	70.9%	261,209 (224,989 to 285,068)	346,141 (291,583 to 383,752)	437,884 (361,105 to 495,521)	67.6%	10,606,570 (8,712,705 to 12,596,443)	13,328,885 (10,953,875 to 15,786,409)	16,647,466 (13,503526 to 20,036,302)	57%
	Age-standardized rate (per 100,000)	113.1 (97.3–122.7)	109.3 (91.9–120.3)	99.5 (82.9–112.1)	−11.9%	106.2 (76– 142.9)	101.1 (71.9–136.1)	107.3 (75.9–144.8)	1%	6.0 (5.2–6.6)	6.0 (5.0–6.7)	5.7 (4.7–6.5)	-4.7%	219.3 (179.9–260.8)	210.3 (172.6–248.6)	206.8 (167.9–248.7)	−5.7%
Female	All ages number	2,306,120 (1,948,987–2,548,592)	2,872,443 (2,394,620–3,193,474)	3,510,903 (2,830,142–4,051,879)	52.2%	2,944,588 (2,115,519–3,957,410)	3,680,204 (2,635,265–4,964,649)	5,145,684 (3,682,031–6,958,472)	74.8%	130,917 (109,902–146,631)	172,718 (140,370–195,213)	228,298 (177,697–266,439)	74.4%	5,250,708 (4,259,424–6,378,375)	6,552,647 (5,296,861–7,934,627)	8,656,587 (6,935,384–10,586,101)	64.9%
	Age-standardized rate (per 100,000)	88.8 (75.2–98.4)	84.6 (70.4–94.3)	80.3 (64.8–92.7)	−9.6%	114.2 (82.1–153.6)	108.8 (77.9–146.8)	117.6 (84.2–159.0)	3%	5.3 (4.5– 6.0)	5.3 (4.3–6.0)	5.2 (4.0–6.1)	−-2.6%	203.0 (164.5–246.9)	193.4 (156.2–234.1)	197.9 (158.5–242.0)	−2.5%
Male	All ages number	3,257,624 (2,767,698–3,549,531)	4,151,301 (3,382,690–4,601,402)	4,516,046 (3,712,923–5,108,160)	38.6%	2,098,238 (1,483,991–2,836,979)	2,624,937 (1,848,570–3,548,384)	3,474,834 (2,450,231––7,952)	65.6%	130,293 (112,418–141,166)	173,424 (144,785–191,590)	209,587 (173,630–236,460)	60.9%	5,355,862 (4,407,897–6,231,815)	6,776,239 (5,494,507–7,915,678)	7,990,880 (6,480,003–9,429,640)	49.2%
	Age-standardized rate (per 100,000)	137.5 (117.7–149.1)	134.6 (110.3–148.6)	119.3 (98.3– 134.8)	−13.2%	93.9 (66.3–126.5)	89.9 (63.3–121.4)	93.5 (65.8–126.7)	−0.5%	6.8 (5.9–7.3)	6.8 (5.7–7.4)	6.3 (5.3 –7.1)	−6.2%	231.4 (191.9–269.3)	224.4 (183.5–261.6)	212.7 (173.1–250.9)	−8.1%

Data in parentheses are 95% Uncertainty Intervals (95% UIs). ^a^Percent change from 2000 to 2019.

DALYs, disability-adjusted life years; YLDs, years lived with disability; YLLs, years of life lost.

**Figure 2:**
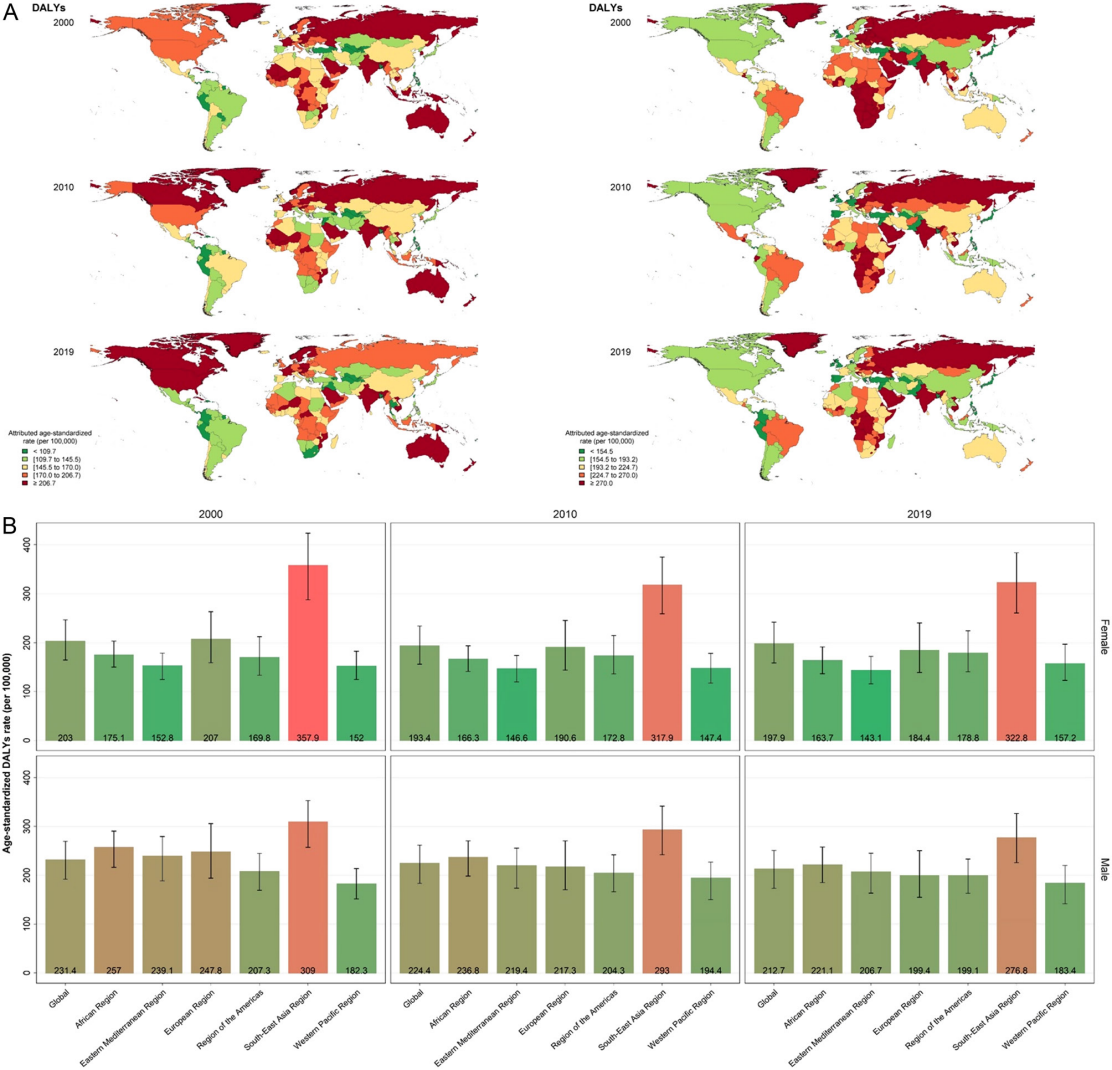
(A) World distribution of DALYs for low BMD at the country level in women (left) and men (right). (B) World distribution of DALYs for low BMD by WHO region in women (up) and men (down).

**Figure 3:**
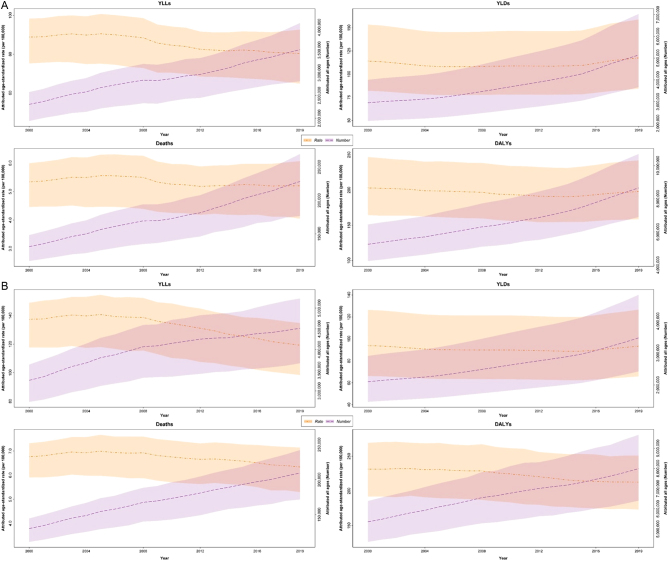
Global all-ages number and age-standardized rate of low bone mineral density burden during 2000–2019 in (A) women and (B) men.

Globally, the number of DALYs was higher in men compared with women in 2000 (5.4 vs 5.25 million) and 2010 (6.8 vs 6.5 million) but lower in 2019 (8.0 vs 8.7 million) and showed a global increase in both sexes during the two decades (percent change of 49.2% and 64.9%, respectively, in men and women; Table 1). However, the age-standardized rate of DALYs was higher in men compared with women at the three points of time (2000: 231.4 (191.9–269.3) vs 203.0 (164.5–246.9); 2010: 224.4 (183.5–261.6) vs 193.4 (156.2–234.1); 2019: 212.7 (173.1–250.9) vs 197.9 (158.5–242.0) per 100,000) and a decreasing trend (though not significant) in the age-standardized DALY rate was observed at the global level in both sexes (percent change of −5.7%), especially men (−8.1% change). World distribution of DALYs attributable to low BMD at a country level and by WHO region is shown in Fig. 2 and the global trend in both numbers and the age-standardized rate is shown in Fig. 3.

#### Years lived with disability

YLDs attributable to low BMD in both sexes increased from 5.0 million (3.6–6.8) in 2000 to 8.6 million (6.1–11.6) in 2019 at the global level (percent change of 71%; Table 1). However, the age-standardized rate of global YLDs did not differ significantly and was 106.2 per 100,000 in 2000 and 107.3 in 2019 (percent change of 1%; Table 1).

Globally, the number of YLDs was higher in women compared with men during 2000 (2.9 vs 2.1 million) to 2019 (5.1 vs 3.5 million) and showed a global increase in both sexes during the two decades (percent change of 74.8% and 65.6%, respectively, in women and men; Table 1). About 60% of the burden was attributed to women.

#### Years of life lost

YLLs attributable to low BMD in both sexes increased from 5.6 million (4.8–6.0) in 2000 to 8.0 million (6.7–9.0) in 2019 at the global level (percent change of 44%; Table 1). However, the age-standardized rate of global YLLs decreased from 113.1 (97.3–122.7) per 100,000 in 2000 to 99.5 (82.9–112.1) in 2019 (percent change of −11.9%; Table 1).

Globally, the number of YLLs was higher in men compared with women during 2000 (3.3 vs 2.3 million) to 2019 (4.5 vs 3.5 million) and showed a global increase in both sexes, especially women during the two decades (percent change of 52.2% and 38.6%, respectively, in women and men; Table 1).

#### Mortality

Mortality attributable to low BMD in both sexes increased from 261,000 (225–285) in 2000 to 438,000 (361–495) in 2019 at the global level (percent change of 67.6%; Table 1). However, the age-standardized rate of global deaths showed a decreasing pattern from 6 (5.2–6.6) per 100,000 in 2000 to 5.7 (4.7–6.5) in 2019 which was not significant (percent change of −4.7%; Table 1).

Globally, the number of deaths was almost the same in women and men in 2000 (130,000) and higher in women compared with men in 2019 (228 vs 209,000) and showed a global increase in both sexes, especially women during the two decades (percent change of 74.4% and 60.9%, respectively, in women and men; Table 1).

#### Sex differences

Regarding the attributable burden in different sexes, while the global YLDs rates were higher in women compared to men (in 2019: 117.6 (84.2–159.0) vs 93.5 (65.8–126.7); Table 1), the attributed YLLs, DALYs, and deaths were higher in men as shown in Table 1 and visualized in Supplementary Fig. 2. YLLs attributable to low BMD were considerably higher in men compared with women globally (in 2019: 119.3 (98.3 –134.8) vs 80.3 (64.8–92.7)) and in all WHO regions except the Southeast Asia region (in 2019: 165.8 (132.4–198.5) vs 170.9 (129.1–207.9)). The least difference between men and women regarding the attributed burdens was observed in Southeast Asia, while the most discrepancy was seen in Africa and EMR. DALYs and deaths attributable to low BMD were relatively higher in men, except for the Southeast Asia region. YLDs were not significantly different between men and women in any region; however, the mean rate was lower in men (Table 2).

**Table 2:** Burden of low bone mineral density by WHO region in 2000, 2010, and 2019 with percent change from 2000 to 2019.

Sex	WHO Region	YLLs					YLDs					Deaths					DALYs					SEV /rank
		Age-standardized rate (per 100,000)			Percent change (%)		Age-standardized rate (per 100,000)			Percent change (%)		Age-standardized rate (per 100,000)			Percent change (%)		Age-standardized rate (per 100,000)			Percent change (%)		
		2000	2010	2019	Change in number^a^	Change in rate^b^	2000	2010	2019	Change in number^a^	Change in rate^b^	2000	2010	2019	Change in number^a^	Change in rate^b^	2000	2010	2019	Change in number^a^	Change in rate^b^	2019
Both	African	155.7 (133.4–174.8)	139.4 (118.8–158.7)	125.8 (106.2–145.6)	38.3%	−19.2%	60.4 (44.0–79.3)	61.5 (44.6–80.7)	65.7 (47.9–86.5)	91%	8.6%	8.0 (6.9–8.9)	7.4 (6.4–8.4)	6.9 (5.9–7.9)	45.9%	−13.2%	216.1 (185.2–244.8)	200.8 (170.3– 229.4)	191.5 (162.8–221.1)	52.5%	−11.4%	23.1 (17–30.2)/ 1
	Eastern Mediterranean	136 (108.9 –155.8)	122.1 (98.3–139.4)	110.5 (87.2–131.7)	53.1%	−18.8%	61.8 (44.3–82.4)	62.4 (44.2–83.8)	66.0 (46.8–89.1)	99.5%	6.8%	6.1 (5.0–7.1)	5.6 (4.6– 6.4)	5.1 (4.2– 6.1)	52.6%	-16.7%	197.9 (161.3–228.4)	184.6 (151.4–215.7)	176.5 (142.4–209.2)	66.8%	−10.8%	15.2 (10.4–21.7)/ 4
	European	85.3 (71.3–93.4)	69.2 (57.5–76.2)	60.3 (49.6– 67.3)	−2.5%	−29.4%	143.8 (100.1–196.9)	135.8 (94.1–187.4)	132.7 (92.0–183.3)	24.7%	-7.7%	4.8 (4.0–5.3)	4.2 (3.4–4.7)	3.8 (3.1 –4.3)	23.6%	-20%	229.1 (177.9–286.8)	205 (157.3–259.6)	192.9 (147.1–246.3)	14.7%	−15.8%	11.7 (7.6–17.6)/ 6
	Region of the Americas	84.6 (71.9 to 91.4)	83.4 (70.7 to 90.8)	80.1 (67.7 to 88.1)	50.9%	−5.3%	104.8 (73.9 to 143.7)	105.8 (74.5 to 144.5)	109.6 (76.8 to 150.6)	73.1%	4.6%	4.5 (3.8 to 4.9)	4.7 (4.0 to 5.2)	4.6 (3.9 to 5.2)	74.8%	3.3%	189.4 (152.5 to 229.7)	189.2 (152.0 to 229.0)	189.7 (152.7 to 230.3)	63.1%	0.2%	14.7 (9.9 to 21.1)/ 5
	South-East Asia	211.3 (169.5 to 239.7)	179.6 (150 to 201.6)	170.0 (139 to 197.7)	57%	−19.6%	125.1 (91.4 to 165.8)	128.9 (93.7 to 170.9)	133.5 (96.9 to 177.3)	100%	6.7%	12.8 (10 to 14.7)	10.7 (8.9 to 12.1)	10.6 (8.5 to 12.3)	77.4%	-17.3%	336.4 (279.1 to 389)	308.6 (257.4 to 360.9)	303.4 (249.2 to 357.2)	73.6%	−9.8%	17.6 (12.5 to 23.9)/ 3
	Western Pacific	90.8 (78.1 to 103.4)	102.8 (76.8 to 117.7)	84.3 (61.2 to 100.1)	57.9%	−7.1%	77.8 (55.5 to 104.4)	68.8 (48.3 to 93.1)	87.0 (61.0 to 118.8)	104.5%	11.8%	4.7 (4.0 to 5.2)	5.5 (3.9 to 6.4)	4.9 (3.3 to 5.9)	100.6%	4.6%	168.6 (139.6 to 197.3)	171.6 (135.6 to 200.5)	171.3 (134.9 to 206.6)	78.7%	1.6%	17.6 (12.5 to 24.1)/ 2
Female	African	113.0 (95.5 to 133.0)	103.5 (87.4 to 124.8)	96.1 (80.5 to 117.1)	48.1%	−15%	62.1 (45.3 to 81.4)	62.7 (45.8 to 82.3)	67.6 (49.5 to 88.8)	96.3%	8.9%	6.5 (5.4 to 7.6)	6.2 (5.2 to 7.3)	5.9 (4.9 to 7.1)	56.1%	−9.3%	175.1 (150.0 to 203.3)	166.3 (141.4 to 193.6)	163.7 (136.6 to 191.4)	65.1%	−6.5%	28.8 (22 to 36.3)/ 1
	Eastern Mediterranean	89.7 (73.2 to 105.0)	82.2 (66.6 to 96.4)	73.7 (59.5 to 87.7)	51.7%	−17.8%	63.1 (45.5 to 83.9)	64.4 (46.1 to 86.4)	69.3 (49.4 to 93.5)	104.9%	9.9%	4.6 (3.6 to 5.7)	4.3 (3.4 to 5.3)	3.9 (3.1 to 4.8)	53.8%	−14.9%	152.8 (124.9 to 178.7)	146.6 (119.7 to 173.9)	143.1 (115.8 to 172.1)	73.2%	−6.4%	18.2 (12.9-24.6)/ 5
	European	60.8 (51.0 to 66.8)	49.3 (40.9 to 54.8)	43.6 (36.0 to 49.2)	1.4%	−28.3%	146.2 (102.3 to 200.1)	141.2 (98.5 to 194.2)	140.7 (97.8 to 194.5)	27.2%	-3.7%	4.0 (3.3 to 4.6)	3.5 (2.8 to 4.0)	3.2 (2.5 to 3.7)	21.6%	−20.8%	207.0 (159.0 to 263.5)	190.6 (144 to 245.5)	184.4 (139.2 to 240.6)	19.6%	−10.9%	14.7 (9.9-21)/ 6
	Region of the Americas	57.1 (48.6 to 62.2)	57.8 (48.8 to 63.4)	55.9 (47.0 to 61.9)	59%	−2.1%	112.8 (79.4 to 154)	115.0 (81.0 to 156.9)	122.9 (86.5 to 167.9)	77%	9%	3.6 (3.0 to 4.0)	3.9 (3.2 to 4.4)	3.8 (3.1 to 4.4)	79.2%	6.7%	169.8 (133.6 to 212.3)	172.8 (136.2 to 214.7)	178.8 (140.5 to 224.6)	70.9%	5.3%	18.6 (13.1 to 25.5)/ 4
	South-East Asia	216.8 (161.3 to 256.9)	174.7 (140.0 to 203.5)	170.9 (129.1 to 207.9)	65.1%	−21.2%	141.0 (103.3 to 186.8)	143.2 (104.7 to 190.1)	151.9 (111.3 to 201.4)	114.1%	7.7%	14.0 (10.2 to 16.9)	11.2 (8.8 to 13.2)	11.3 (8.6 to 13.8)	85.6%	−19.3%	357.9 (287.9 to 423.6)	317.9 (259.2 to 375.5)	322.8 (260.8 to 384.2)	85.4%	−9.8%	22.9 (17.1 to 29.7)/ 2
	Western Pacific	66.7 (57.3 to 74.3)	73.4 (54.7 to 84.8)	62.8 (41.8 to 77.9)	72.1%	−5.8%	85.3 (60.9 to 114.8)	74.0 (52.2 to 100.0)	94.4 (66.0 to 128.5)	106.7%	10.6%	4.0 (3.3 to 4.4)	4.6 (3.1 to 5.5)	4.2 (2.6 to 5.3)	121.3%	6.6%	152.0 (124.7 to 182.7)	147.4 (117.4 to 178.1)	157.2 (122.9 to 197.0)	91.4%	3.4%	22.5 (16.7 to 29.1)/ 3
Male	African	199.1 (166 to 224.8)	177.4 (147.1 to 205.1)	158.3 (129.8 to 188)	33.1%	−20.5%	57.9 (41.9 to 76.1)	59.4 (42.6 to 78.0)	62.8 (45.1 to 82.6)	85.4%	8.4%	9.6 (8.1 to 10.8)	8.9 (7.4 to 10.3)	8.1 (6.8 to 9.7)	38.9%	−15.3%	257.0 (216.1 to 290.3)	236.8 (198.4 to 270.6)	221.1 (185.0 to 257.9)	44.3%	−14%	16.8 (11.5 to 23.8)/1
	Eastern Mediterranean	178.5 (132.9 to 209.3)	158.8 (119.9 to 184.8)	143.8 (109.0 to 174.3)	53.7%	−19.4%	60.6 (43.1 to 81.2)	60.6 (42.9 to 81.5)	62.9 (44.3 to 85.5)	94.5%	3.8%	7.5 (5.9 to 9.0)	6.7 (5.4 to 7.9)	6.2 (4.9 to 7.5)	52%	−17.7%	239.1 (188.5 to 279.2)	219.4 (173.5 to 255.7)	206.7 (163.4 to 245.0)	63.4%	−13.6%	12.4 (7.7-18.8)/ 2
	European	112.6 (93.8–122.4)	92.3 (77.0–100.6)	79.6 (65.6––89.5)	-5.6%	−29.3%	135.2 (93.5189.9)	125.0 (86.4175.4)	119.8 (82.8–168.5)	20.7%	-11.4%	5.7 (4.8––	5.1 (4.3–5.6)	4.7 (3.9–5.2)	26%	17.5%	247.8 (194.2 to 305.7)	217.3 (170.5–270.4)	199.4 (154.9–250.3)	8.8%	−19.5%	8 (4.3–13.4)/ 6
	Region of the Americas	114.9 (97.9–123.4)	111.4 (94.1–120.3)	106.7 (90.8–117.5)	46%	−7.1%	92.4 (64.9–127)	92.9 (65.2–127.2)	92.3 (64.9–127.3)	66.9%	-0.1%	5.6 (4.8–6.0)	5.7 (4.9–6.2)	5.6 (4.8–6.1)	70.7%	0.3%	207.3 (169.1–244.8)	204.3 (166.2–241.9)	199.1 (163.2–233.4)	55%	−4%	10.2 (6–16.4)/ 5
	South-East Asia	201.9 (164.8–228.1)	181.4 (149.2–204.0)	165.8 (132.4–198.5)	49.3%	−17.8%	107.1 (77.0–142.2)	111.6 (80.3–148.6)	111.0 (79.7–147.8)	83.1%	3.6%	11.3 (8.9–12.8)	9.9 (8.3–11.2)	9.5 (7.7–11.4)	67.5%	−15.2%	309.0 (257.2–352.9)	293.0 (242.1– 341.6)	276.8 (226.0– 326.5)	61.4%	−10.4%	11.3 (7–17.6)/ 4
	Western Pacific	114.5 (97.5– 136.0)	132.4 (97.1– 153.8)	106.1 (76.5–131.5)	49.5%	−7.3%	67.8 (47.9– 91.9)	62.0 (43.6– 84.4)	77.3 (54.3–105.7)	101.6%	14%	–5.4 (4.76.2)	6.4 (4.7–7.5)	5.6 (4.0–6.9)	83.1%	3.2%	182.3 (151.8–213.7)	194.4 (150.1–227)	183.4 (141.7–220.2)	67.6%	0.6%	12.1 (7.5–18.7)/ 3

Data in parentheses are 95% uncertainty intervals (95% UIs). ^a^Percent change in all-age number from 2000 to 2019. ^b^Percent change in age-standardized rate from 2000 to 2019.

DALYs, disability-adjusted life years; YLDs, years lived with disability; YLLs, years of life lost.

#### Time trend

The greatest change observed globally from 2000 to 2019 was for YLDs number in both women (74.8%) and men (65.6%) followed by death (74.4% and 60.9%, respectively) (Table 1). Regarding WHO regions, the greatest percent change for YLD was observed in the Western Pacific region (104.5%) followed by Southeast Asia (100%), and the change was greater in women in all regions (Table 2). As for age-standardized rates, the greatest reduction was observed in the global YLL rate (percent change of −11.9%; Table 1), followed by DALYs (−5.7%). Most of the reduction in the burden rate was observed in men compared with women (e.g., 13.2% vs 9.6% for YLL; Table 1). The reduction in the burden rate was mostly observed in the European region (29.4%, 20%, 15.8%, and 7.7% for YLL, death, DALY, and YLD rates, respectively), and Europe was the only region showing YLD rate reduction (Table 2).

#### WHO regions

**Figure 4:**
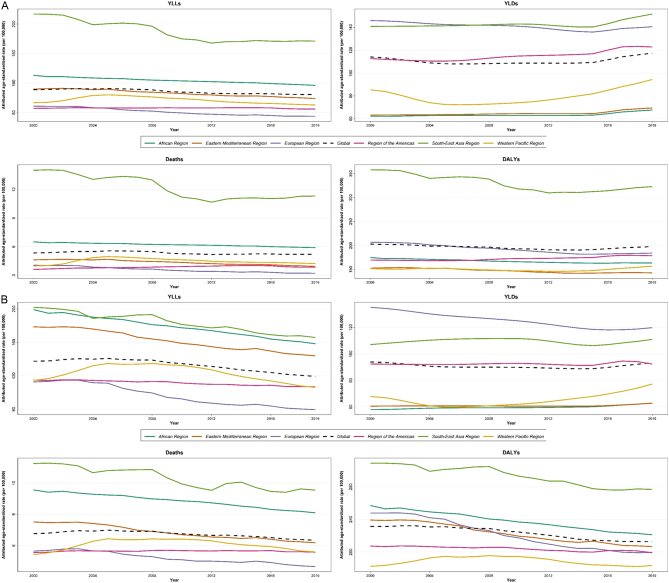
Age-standardized attributed burden rate of low bone mineral density by WHO region from 2000 to 2019 in (A) women and (B) men.

Overall, the highest DALYs, YLLs, and death rates were observed in women and men of the Southeast Asia region. While the European region showed the highest YLDs in men (119.8 (82.8–168.5) in 2019) and until the recent decade in women, this region showed the lowest YLL (60.3 (49.6–67.3) in 2019) and death rate (3.8 (3.1–4.3) in 2019) in both women and men (Fig. 4). The trend in the age-standardized burden rate of low BMD in different WHO regions from 2000 to 2019 in women and men is shown in Fig. 4.

#### Age groups

**Figure 5:**
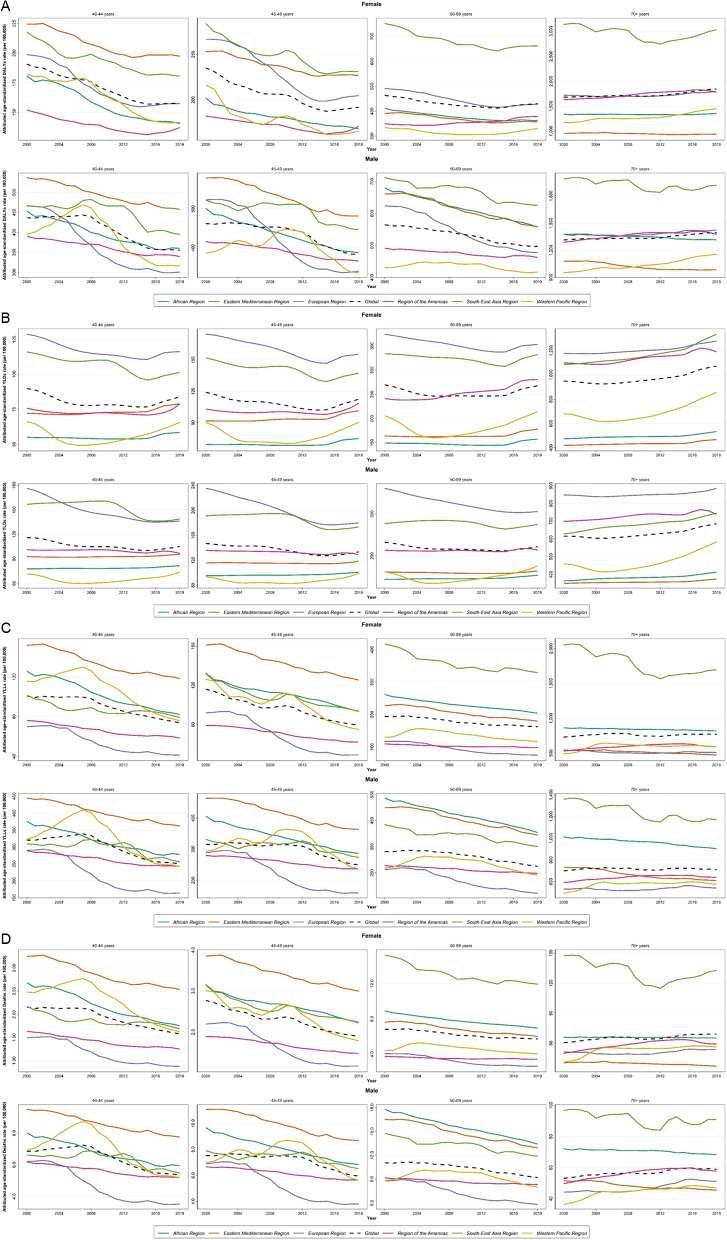
The attributed burden of low bone mineral density in WHO regions by age group during 2000–2019. (A) DALYs, (B) YLDs, (C) YLLs, and (D) deaths.

Global DALYs rates were near 2000 and 1500 per 100,000 in women and men over 70 years, about 500 in those 50–70 years, and less than 250 in those 40–50 years. As observed, the population over 70 years accounts for most of the attributable burden (Fig. 5), and the highest DALYs, YLLs, and deaths in this age group are related to the Southeast Asia region, while the highest YLDs are observed in the European region in both women and men. Almost the same trend in all indices is observed in women 50–70 years. In 50- to 70-year-old men, the same trend is observed for DALYs and YLDs, while the highest YLLs and mortality are seen in the African region (Fig. 5). Individuals younger than 50 years followed a different pattern and EMR showed the highest burden regarding DALY, YLL, and death compared to other regions, while the European region showed the highest YLD similar to older individuals. While in the age group of more than 70 years, higher age-standardized rates of burden were observed in women, in the age group of less than 50 years, the rates were higher in men (Fig. 5, notice the values in the *y*-axis).

## Discussion

According to the GBD study, the global age-standardized SEV of low BMD is estimated to be 20.7% in women and 11.3% in men in 2019. Among the WHO regions, Africa had the highest age-standardized SEV of low BMD in women (28.8%) as well as in men (16.8%), followed by Southeast Asia (22.9%), and Western Pacific (22.5%) in women, and EMR (12.4%) and Western Pacific (12.1%) in men. The lowest SEV was observed in Europe in both women (14.7%) and men (8%). The age-standardized data showed an improving trend in the global burden rate of low BMD, especially YLLs and DALYs which showed an 11.9% and 5.7% reduction from 2000 to 2019; however, the absolute numbers showed an about 70% increase in the YLDs and deaths attributed to low BMD from 2000 to 2019 at the global level.

The observation in the SEV of low BMD can be interpreted to be partly in line with the report of a recent systematic review and meta-analysis on the worldwide prevalence of osteoporosis which was 23.1% in women and 11.7% in men, with the highest prevalence (39.5%) observed in Africa (15). The prevalence of osteoporosis in the EMR was 20.5% in men and 24.4% in women and with the highest prevalence observed in Saudi Arabia and showed an increasing trend through 2015, indicating a considerable prevalence, especially in men (16). The prevalence, however, increases with aging, and in Iran, for example, the age-standardized prevalence of osteoporosis was estimated to be as high as 62.7% in women and 24.6% in men aged ≥60 years (17) and the condition affects more than one-third of people aged 50 years and older in China (18). The distribution of osteoporosis varies across different populations so as the fragility fractures. A worldwide study reported that the incidence of vertebral fractures in both sexes rises with aging above 50 years and in any given country is higher in women than in men. The variability observed in vertebral fracture prevalence between different countries is low in Europe and North America; however, greater variations have been observed in Asia and Latin America (19).

The increase in the absolute numbers of the burden of low BMD despite the improving trend in the global burden rate can reflect the population aging. Compared with other risk factors, low BMD had a lower contribution to the global burden (12), which may not seem to be supported by the findings of other studies regarding the significant burden of hip fractures (6, 7, 20). The low contribution could be an underestimation partly caused by the definition of low BMD in GBD studies (21) as well as a lack of data on osteoporosis and fragility fractures as diseases and injuries. In clinical settings, the diagnosis of osteoporosis is based on the lowest DXA T-score of the lumbar spine, femoral neck, or total hip according to the National Osteoporosis Foundation (22) and the International Society for Clinical Densitometry (ISCD) classification (23); those with the lowest T-score ≤−2.5 are diagnosed to have osteoporosis, those with the lowest T-score ≥−1.0 are normal, and those in between are considered as osteopenia or low bone mass. BMD of young Caucasian females is used as a reference in both women and men, which is reasonable in the prediction of absolute fracture risk and diagnosis of osteoporosis, since each unit decreases BMD and increases the risk of fracture and it is same in men and women (3). In the GBD study, however, low BMD was defined in terms of the difference between the BMD of a population and the 99th percentile of a reference population using age- and sex-specific TMREL, because this study focuses on modifiable risk factors rather than the inevitable bone loss caused by aging. This could lead to underestimation of exposure in older ages while using the WHO definition and ISCD guidelines result in higher prevalence estimates.

Despite the highest SEVs observed in the African region, the highest DALYs, YLLs, and deaths were observed in women and men of the South-East Asia Region. However, the European region showed the highest YLDs in men and until the recent decade in women. The discrepancy can be explained in part by the difference in life expectancy, quality of life, and access to healthcare services in different regions. Also, peak bone mass (PBM) that is attained in adolescence and early adulthood is related to the risk of developing osteoporosis and individuals who achieve higher PBM have a lower risk of osteoporotic fractures and have to lose more bone mass before reaching a critical threshold associated with osteoporosis. Sex, genetics, sex hormones, nutrition, physical activity, and lifestyle factors all affect PBM acquisition (24) which can explain the differences between the prevalence and burden of low BMD in different sexes and different regions.

The population over 70 years, especially women, account for most of the attributable burden when compared with those 50–70 years and younger, which is expected according to the degenerative nature of osteoporosis. As the population ages over 50 years, the prevalence of osteoporosis increases from 10% at age 60 to as many as 65% at age 90 years in women (20). The same increasing trend is observed in the risk of fragility fractures. After menopause, rapid bone loss occurs in women due to reduced estrogen levels, followed by age-related bone loss in elderly women and men. However, in the population less than 50 years, generally the age-standardized rate of burden is higher in men, which could be explained by the protective effect of estrogen in pre-menopausal women, and riskier lifestyle habits in men of this age group.

In this study, we visualized the current state of exposure to low BMD, the attributable burden globally and in different WHO regions, and the trend from 2000 to 2019 according to the GBD study 2019. Besides, we compared different regions, age categories, and sexes. The main limitation of the present report is that we didn’t have direct access to data on the exposure and burden of osteoporosis and fragility fractures as diseases and injuries in the GBD study (12); besides, the definition of low BMD was not the same as what we use in clinical practice; the TMREL used for low BMD and other risk factors may mask the effect of age and sex. BMD at the femoral neck was used, and the other sites were not considered. These all could underestimate the prevalence of clinically low BMD and the burden of fragility fractures.

Genetic variations and ethnic differences; nutritional issues like inadequate intake of calcium, vitamin D, and other essential nutrients necessary for bone health, as well as poor dietary habits and limited access to nutritious food; cultural practices affecting dietary choices, sedentary lifestyles, and lack of physical activity and insufficient weight-bearing exercise during childhood and adolescence (affecting PBM); and socioeconomic disparities such as poverty, limited access to healthcare and educational facilities, and some cultural habits like smoking and excessive caffeine intake are all factors contributing to lower BMD and the attributable burden. Policymakers should address these issues, especially in regions of high SEV and burdens by public health education and raising awareness about the risk factors of low BMD and the prevention strategies. They should target both the general population and healthcare professionals and focus on the awareness about and access to healthy food options, enhancing regular physical activity in school and workplace, healthcare infrastructure, and policies to control smoking. Specific interventions are based on the cultural, socioeconomic, and healthcare context of each region.

## Conclusion

Our findings support the considerable global exposure to low BMD, especially in Africa and in women. An improving trend in the global rate of YLLs, DALYs, and deaths was visualized, mostly in the European region. The results of this study also reflect that the burden attributed to low BMD in younger individuals, especially men, is critical in the EMR, which requires urgent attention and appropriate actions. Knowledge of the burden attributable to low BMD in different regions can increase the awareness of healthcare decision-makers to identify priorities and adopt appropriate strategies for the early screening, diagnosis, and treatment of the condition and also strategies to prevent falls and fragility fractures and their consequent morbidity and mortality.

## Supplementary Materials

Supplementary Figure 1. Age-standardized summary exposure value to low bone mineral density by WHO region from 2000 to 2019 in women (up) and men (down)

Supplementary figure 2. Global age-standardized attributed burden of low bone mineral density by sex during 2000-2019

## Declaration of interest

The authors declare that they have no competing interests.

## Funding

This work did not receive any specific grant from any funding agency in the public, commercial, or not-for-profit sector.

## Consent for publication

Not applicable.

## Ethics approval and consent to participate

Not applicable.

## Availability of data and materials

All data generated or analyzed during this study are included in this published article (and its supplementary information files).

## Author contribution statement

Contributors, N P, S S M, and N F designed the study. S S M, N F, N R, and A O analyzed the data. N P, M S, M M R, P S, and B L, drafted the manuscript. All authors contributed to the interpretation of the data and revision of the manuscript. All authors have read and agreed to the published version of the manuscript.
