# Supplementary figures and images for: Trend in global burden attributable to low bone mineral density in different WHO regions: 2000 and beyond, results from the Global Burden of Disease (GBD) study 2019
Source: Endocr Connect. 2023 Sep 14;12(10):e230160. doi: 10.1530/EC-23-0160 (PMC10503222; doi:10.1530/EC-23-0160)

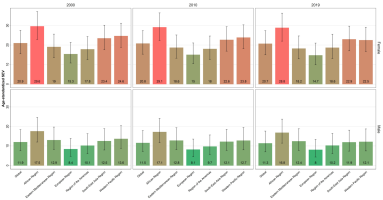

Supplement: Supplementary Figure 1. Age-standardized summary exposure value to low bone mineral density by WHO region from 2000 to 2019 in women (up) and men (down) [file supplementary_figure_1.pdf]

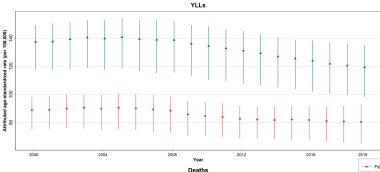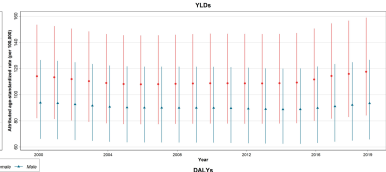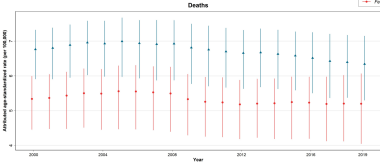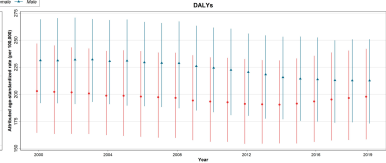

Supplement: Supplementary figure 2. Global age-standardized attributed burden of low bone mineral density by sex during 2000-2019 [file supplementary_figure_2.pdf]
